# Combining vestibular rehabilitation with noisy galvanic vestibular stimulation for treatment of bilateral vestibulopathy

**DOI:** 10.1007/s00415-022-11033-x

**Published:** 2022-02-25

**Authors:** Josefine Eder, Silvy Kellerer, Tamara Amberger, Aram Keywan, Julia Dlugaiczyk, Max Wuehr, Klaus Jahn

**Affiliations:** 1 German Center for Vertigo and Balance Disorders (DSGZ), Ludwig-Maximilians University of Munich (LMU), University Hospital Grosshadern, Marchioninistrasse 15, 81377 Munich, Germany; 2 Department of Neurology, Schoen Clinic Bad Aibling, Bad Aibling, Germany; 3 Clinic for Ear, Nose, Throat and Facial Surgery, Interdisciplinary Center for Vertigo and Neurological Disorders, University of Zurich, Zurich, Switzerland

**Keywords:** Bilateral vestibulopathy, Vestibular rehabilitation, Noisy galvanic vestibular stimulation, Balance, Gait

## Abstract

**Objective:**

Noisy galvanic vestibular stimulation (nGVS) has been shown to partly restore vestibular function and to stabilize stance and gait in patients with incomplete bilateral vestibulopathy (BVP). Here, we examined potential synergistic effects of nGVS when combined with standardized vestibular rehabilitation training (VRT).

**Methods:**

23 patients with confirmed BVP received a 30-min vestibular rehabilitation training (VRT) program three times a week for 2 weeks. The intervention group (*n* = 12) was stimulated with nGVS (at individually determined optimal amplitudes) during training, whereas the control group (*n* = 11) received zero-amplitude nGVS (sham stimulation) during training. Outcome measurements assessed at baseline, after 2 weeks of training, and at 2-week follow-up included quantitative posturography, instrumented gait analysis, Timed Up and Go Test (TUG), Functional Gait Assessment (FGA), and clinical scores related to quality of life and balance confidence.

**Results:**

After 2 weeks of VRT, all patients showed moderate improvement in balance. Irrespective of nGVS treatment, performance improved in the TUG (*p* < 0.013), and in the FGA (*p* < 0.040). Furthermore, base of support when walking with closed eyes was reduced after 2-week training (*p* < 0.003). Postural sway did not change. There was no difference between groups and thereby no evidence for an additional influence of nGVS on the VRT treatment effects.

**Conclusion:**

nGVS does not induce synergistic treatment effects in combination with VRT in patients with BVP when applied during treatment sessions. Hence, rather than being applied in parallel, nGVS and VRT might be complementary therapeutic options with nGVS being used during postural activities in daily life, e.g., walking.

## Introduction

Patients with bilateral vestibulopathy (BVP) suffer from a complete or incomplete loss of function of peripheral vestibular structures, presenting with chronic dizziness including postural imbalance when standing or walking, especially in darkness or on uneven ground. Head or body movements cause visual blurring or oscillopsia, i.e., illusionary bouncing of the visual scene, which causes difficulties in gaze stabilization and keeping one’s balance [23]. In most patients, this results in a lower quality of life and a higher risk of falls [24]. The only therapeutic option currently available to sufficiently improve outcome for BVP patients is vestibular rehabilitation therapy (VRT), which aims to improve balance by the training of multisensory postural control to compensate and substitute the vestibular hypofunction [9]. However, the long-term effects of this intervention are limited [21].

Recently, white noise galvanic vestibular stimulation (nGVS) at imperceptible levels was used to modify vestibular perception and performance [5, 28]. When an optimal amplitude of noise is added to a nonlinear system, such as the vestibular system, a mechanism known as stochastic resonance is hypothesized to enhance the ability to detect and process weak signals [19] and hence to improve vestibular functions [16]. nGVS transcutaneously delivered to the mastoid processes has been shown to facilitate postural stabilization while standing or walking in healthy subjects as well as in patients with BVP [8, 29, 30]. This makes it a promising non-invasive treatment option for patients with peripheral vestibular hypofunction.

It is not known whether nGVS might induce beneficial synergistic effects when combined with VRT in patients with BVP. Hence, the primary goal of this study was to determine whether the application of nGVS during VRT promotes a better overall recovery than rehabilitation alone (i.e., during sham nGVS) in patients with BVP.

## Methods

This double-blinded clinical explorative study aimed to evaluate the impact of imperceptible amounts of nGVS on the efficacy of vestibular rehabilitation in patients with BVP. The study protocol was approved by the ethics committee of the University of Munich and was conducted in accordance with the Declaration of Helsinki. All participants gave their written informed consent.

### Subjects

Twenty-three patients (9 females, mean age 62.3 ± 14.3 years) participated in this study. All of them showed a clinically proven BVP, confirmed either by bilaterally reduced responsiveness to bithermal (44 and 30 °C) caloric irrigation (mean peak slow-phase velocity < 6 deg/s) or a pathological video head impulse test on both sides (gain < 0.6) [23].

### Noisy galvanic vestibular stimulation

nGVS was delivered via a pair of conductive-rubber electrodes (4.0 cm × 6.0 cm), placed in two saline-soaked sponges, that were attached over the left and right mastoid process behind the participant’s ears. Electrodes were connected to a portable direct current stimulator (neuroConn^®^, Ilmenau, Germany), which delivered the electrical signal consisting of a zero-mean Gaussian white noise within a frequency range of 0–30 Hz. To identify the individual optimal nGVS amplitude, each patient performed eight quiet-standing trials with eyes closed for a duration of 30 s on a stabilometer platform (Kistler 9261A, Winterthur, Switzerland). During each trial, patients received nGVS at a different intensity (from 0 to 700 µA in steps of 100 µA) in a randomized order and their performance during stimulation trials was compared to their baseline performance (i.e., nGVS at 0 µA). Improvement in balance performance due to nGVS was determined based on the three different posturography parameters, i.e., mean velocity, area, and root mean square of sway. The stimulation amplitude at which individual patients exhibited the best improvement in all three parameters was assigned as their ‘optimal nGVS amplitude’. For the sham condition of nGVS, the intensity of the electrical signal was set to 0 µA.

### Vestibular rehabilitation therapy

The rehabilitation program was individually adjusted to the deficits and therapeutic demands of each patient, to ensure that VRT exercises were sufficiently challenging to induce treatment effects [26]. The basic exercises included training of gaze stabilization during standing and walking and eye–head coordination during standing and walking as well as practices to optimize balance strategies. To challenge patients even more, exercises could be performed on foam or with eyes closed. Furthermore, several different tasks were combined with each other or performed while walking. These basic exercises were individually adapted to the main demands of each patient. This way, the VRT program specifically targeted the individual physical problems. Each therapy session was guided and supervised by an expert vestibular physical therapist and lasted 30 min.

### Procedures

**Fig. 1:**
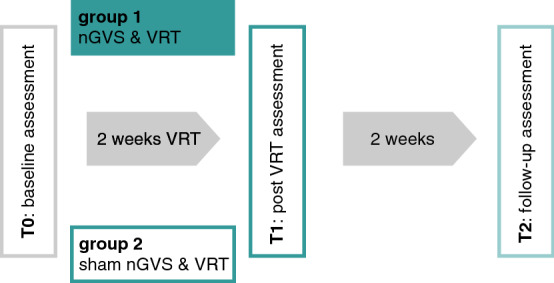
Scheme of the study protocol. Following an initial baseline assessment, patients were randomly assigned to an intervention group (group 1) or a control group (group 2). Group 1 received 2 weeks of VRT with nGVS at optimal intensity, while group 2 received sham stimulation during training. Treatment effects were assessed immediately after 2 weeks of training (T1) and at 2-week follow-up. *nGVS* noisy galvanic vestibular stimulation,* VRT* vestibular rehabilitation therapy

Participants were randomly assigned to one of two groups. The intervention group received nGVS during VRT; the control group received sham stimulation during VRT. In both trial arms, patients were provided with a VRT program three times a week for 2 consecutive weeks (30 min rehabilitation per session). For patients in the intervention group, nGVS was active during all rehabilitation sessions. Patients in the control group received sham nGVS during each rehabilitation session. Outcome measures were assessed at baseline (T0), after 2 weeks of training (T1) and at 2-week follow-up (T2) (see Fig. 1).

### Outcome measures

Several balance and gait tests and questionnaires were applied at the three assessment time points (T0–T2). Primary outcome was postural stability as assessed during posturography while standing on foam with eyes closed. The amount of body sway was quantified by the mean velocity of sway. Secondary outcomes were the patient’s gait performance, mobility, and dynamic balance. Gait assessment was performed on a pressure-sensitive gait mat (GAITRite^®^, CIR System, Sparta, NJ, USA) while walking with eyes closed. Gait performance was evaluated by quantifying walking velocity, base of support, and the coefficient of variation (CV) of stride time. Patients’ functional mobility and dynamic balance were assessed by the Berg Balance Scale (BBS; max. 56) [13], the Functional Gait Assessment (FGA; max. 30) [27], and the Timed Up and Go Test (TUG; times of > 13.5 s are related to an increased risk of falling in older adults) [3]. Additionally, patients completed the following questionnaires: the Dizziness Handicap Inventory (DHI; 16–34 points = mild handicap, 36–52 points = moderate handicap), > 54 points = severe handicap) [13] to evaluate the self-perceived handicap due to dizziness, the International Physical Activity Questionnaire (IPAQ; low, moderate, or high physical activity score) [18] to score the level of physical activity, the Falls Efficacy Scale International (FES-I; max. 64) [10] to assess balance confidence and fear of falling, and the Activities-specific Balance Scale (ABC-d; max. 100%) [13] to assess confidence in performing daily activities.

### Statistical analysis

Data are reported as mean ± SD. Since most of the outcome measures did not exhibit normal distribution, non-parametric tests were performed to assess differences in treatment effects between the intervention and control groups. For all outcome parameters, the Friedman test was used to assess treatment effects between the three assessment time points (T0, T1, T2). Significant treatment effects were subsequently compared between the intervention and control groups using the Mann–Whitney *U* test. Results were considered significant if *p* < 0.05. Statistical analysis was performed using SPSS (Version 26.0, IBM Corp., USA).

## Results

### Characterization of the study cohort

The study cohort consisted of 23 patients with a clinically proven BVP. The random assignment of these patients to either the intervention group or the control group yielded two homogenous cohorts with respect to age and gender distribution, vestibular hypofunction, and balance deficits (see Tables 1, 2). The results of the questionnaires filled by the patients resulted in subjective as well as objective moderate dizziness which did not change significantly during the period of the study (see Table 2).

**Table 1:** Characterization of the study cohorts

	Intervention group	Control group	
Age	61.92 ± 15.93 y	62.64 ± 13.15 y	*p* = 0.926
Gender	4 f/8 m	5 f/6 m	*p* = 0.552
SPV (during caloric irrigation)	5.055 ± 6.854°/s	5.650 ± 3.188°/s	*p* = 0.080
vHIT gain	0.280 ± 0.215	0.379 ± 0.213	*p* = 0.184
Disease duration	6.41 ± 8.85 y	10.33 ± 12.81 y	

*f* female, *m* male, *SPV* slow-phase velocity, *y* years

**Table 2:** Results of assessments

	Intervention group	Control group	
T0—sway velocity (standing on foam, eyes closed)	313.840 ± 204.215 cm/s	556.167 ± 287.200 cm/s	*p* = 0.056
T1—sway velocity (standing on foam, eyes closed)	263.421 ± 166.910 cm/s	501.692 ± 275.694 cm/s	*p* = 0.023
T2—sway velocity (standing on foam, eyes closed)	332.191 ± 171.511 cm/s	550.593 ± 249.715 cm/s	*p* = 0.036
T0—gait velocity (walking with eyes closed)	61.833 ± 26.012 cm/s	63.503 ± 25.271 cm/s	*p* = 0.712
T1—gait velocity (walking with eyes closed)	65.949 ± 26.640 cm/s	66.919 ± 22.080 cm/s	*p* = 0.758
T2—gait velocity (walking with eyes closed)	61.147 ± 26.755 cm/s	65.438 ± 24.439 cm/s	*p* = 0.951
T0—stride time CV (walking with eyes closed)	18.240 ± 13.991%	11.580 ± 7.695%	*p* = 0.097
T1—stride time CV (walking with eyes closed)	16.129 ± 19.920%	10.532 ± 7.052%	*p* = 0.498
T2—stride time CV (walking with eyes closed)	11.350 ± 5.474%	15.126 ± 13.450%	*p* = 1.000
T0—base of support (walking with eyes closed)	20.962 ± 6.117 cm	20.417 ± 7.442%	*p* = 0.667
T1—base of support (walking with eyes closed)	19.000 ± 5.791 cm	19.625 ± 6.258 cm	*p* = 1.000
T2—base of support (walking with eyes closed)	21.556 ± 5.306 cm	20.277 ± 6.277 cm	*p* = 0.389
T0—TUG	7.985 ± 2.676 s	6.947 ± 1.260 s	*p* = 0.468
T1—TUG	7.450 ± 2.892 s	6.292 ± 1.287 s	*p* = 0.391
T2—TUG	7.190 ± 2.515 s	6.412 ± 0.987 s	*p* = 0.356
T0—FGA	23.417 ± 4.963	23.000 ± 3.194	*p* = 0.805
T1—FGA	23.667 ± 4.030	26.091 ± 2.737	*p* = 0.137
T2—FGA	24.917 ± 4.166	25.636 ± 2.942	*p* = 0.877
T0—DHI	40.833 ± 17.837	42.727 ± 14.867	*p* = 0.734
T1—DHI	39.333 ± 21.309	44.000 ± 17.550	*p* = 0.423
T2—DHI	36.667 ± 18.884	43.818 ± 20.851	*p* = 0.423
T0—BBS	49.167 ± 6.645	50.273 ± 4.268	*p* = 0.951
T1—BBS	50.250 ± 6.369	50.818 ± 4.750	*p* = 0.975
T2—BBS	49.750 ± 6.152	51.364 ± 3.982	*p* = 0.733
T0—ABC-d	69.245 ± 26.027	78.693 ± 17.255	*p* = 0.372
T1—ABC-d	75.219 ± 21.443	70.881 ± 22.502	*p* = 0.601
T2—ABC-d	74.542 ± 20.264	76.421 ± 13.406	*p* = 0.902
T0—FES-I	25.000 ± 6.396	25.727 ± 9.717	*p* = 0.805
T1—FES-I	24.083 ± 6.921	26.364 ± 9.233	*p* = 0.557
T2—FES-I	24.333 ± 5.662	26.636 ± 10.957	*p* = 0.902

*CV* coefficient of variation, *TUG* timed up and go test; *FGA* Functional Gait Assessment, *DHI* Dizziness handicap inventory, *BBS* Berg Balance Scale, *ABC-d* Activities-specific Balance Scale, *FES-I* Falls Efficacy Scale International

### General effects of VRT

**Fig. 2:**
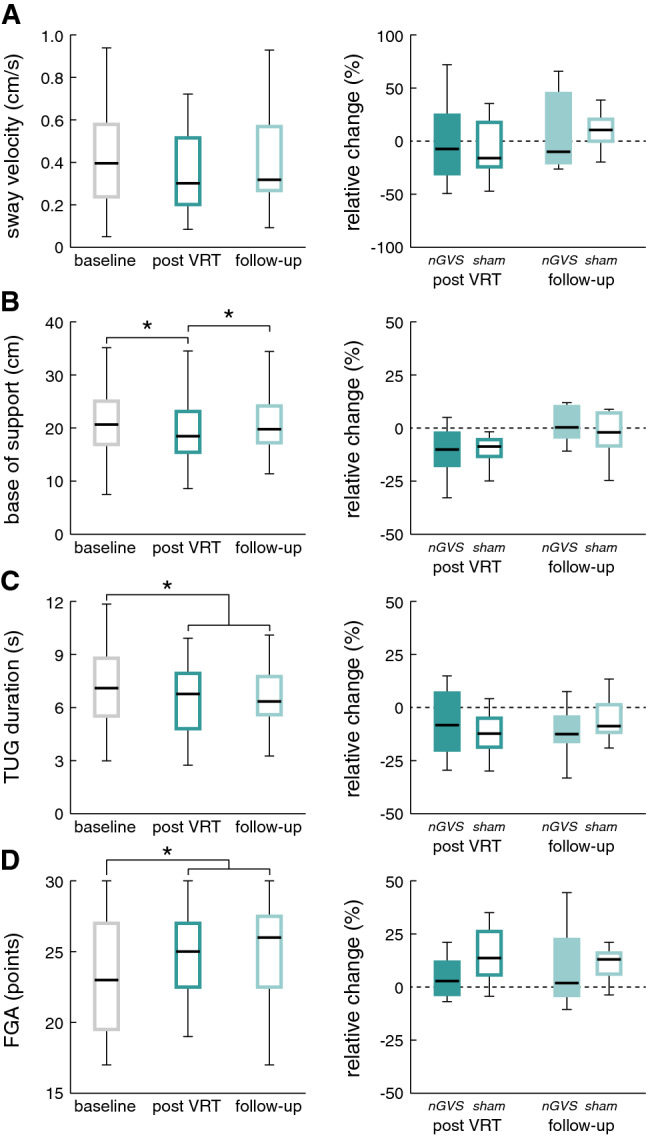
VRT and nGVS treatment effects. Left panel: general VRT-related treatment effects (for the combined intervention and control group) at T1 (post VRT) and T2 (follow-up) compared to T0 (baseline assessment) for **A** sway velocity while standing on foam with eyes closed, **B** base of support while walking with eyes closed, **C** TUG, and **D** the FGA. Right panel: comparison of relative changes in the respective outcome measures between the intervention and control groups. Moderate general treatment effects of VRT were found for gait capacity, TUG and FGA performance, but not for static posturography. Treatment effects did not differ between the intervention and control groups. *VRT* vestibular rehabilitation therapy; *nGVS* noisy galvanic vestibular stimulation, *TUG* timed up and go test, *FGA* functional gait assessment; *Indicates a significant difference

To examine general effects of VRT, we analyzed training effects on each outcome measure at T1 (after 2 weeks of training) and T2 (at 2-week follow-up) compared to baseline T0. Figure 2 shows in the left panel measurements at baseline (T0), post VRT (T1), and at follow-up (T2) for all participants (sham and intervention group). We did not find VRT-induced changes on postural stability measured by posturography, the BBS scale or any of the balance confidence or physical-activity-related questionnaires. In contrast, we found a moderate training effect regarding patients’ gait performance: When walking with closed eyes, the base of support was significantly smaller (*p* < 0.003) at T1 and returned to baseline level at T2 (see Fig. 2, B, left panel). Comparable improvements were further found for tests of functional mobility, i.e., the TUG (*p* < 0.013) and the FGA (*p* < 0.040) that remained stable at follow-up assessment (see Fig. 2C, D, left panel).

### Effects of nGVS

Identification of optimal nGVS intensity yielded an average nGVS amplitude of 330 ± 203 µA. None of the participants felt pain or any other negative symptoms during the application of nGVS. The intervention group received nGVS at optimal intensities during the complete duration of VRT, while the control group received sham nGVS (i.e., 0 µA) during training. Combining nGVS with VRT did not have any effect on the examined outcome measures after 2 weeks of training or after the 2-week follow-up assessment (see Fig. 2, right panel).

## Discussion

Stochastic electrical vestibular stimulation at imperceptible intensities (i.e., nGVS) has been demonstrated to stabilize static posture and walking performance in patients with BVP [6, 11, 22]. Up to now, vestibular rehabilitation (i.e., VRT) is the only established treatment option for patients with BVP [1]. Here, we applied nGVS in addition to a standardized VRT treatment in a placebo-controlled double-blinded clinical study to examine whether the combination of both treatments would yield any synergistic effects. While VRT generally induced moderate improvements in patients’ balance capabilities, we found no evidence that the combination of nGVS with VRT yields any additional effects on either patients' balance capabilities or their subjective balance self-confidence. These results will be discussed with respect to (1) the general effects of vestibular rehabilitation training, (2) the absence of any synergistic effects from nGVS, and (3) considerations on whether and how to use both treatment strategies together in the future.

Two weeks of VRT yielded only moderate balance improvements in patients with respect to clinical tests of functional mobility, i.e., the TUG and FGA, and improved gait performance while walking with closed eyes. However, no effects were found with respect to clinical scores on balance confidence or static posturography—the primary outcome measure of this study. This observation is in contrast to earlier reports which found clear VRT treatment effects on static body sway and related outcome measures [13]. This discrepancy could be due to differences in the specific VRT protocol and/or differences in study cohorts. For instance, VRT in the present study only lasted for 2 weeks, while in previous studies, VRT was often applied for considerably longer periods, which might account for treatment effects in BVP [2, 13, 17]. Another reason for the observed weakness of VRT treatment effects could be that patients in our cohort had a relatively moderate severity of BVP-related symptoms compared to previous studies [3] and might therefore only exhibit weak responses when treated with VRT.

Combining nGVS with VRT did not yield any additional effects on patients’ stance or gait performance or their balance self-confidence. This absence of synergistic effects of nGVS with VRT might be related to the mode of action of imperceptible stochastic vestibular stimulation, which is thought to improve vestibular function by stochastic resonance, a mechanism in which subthreshold sensory signals become enhanced and detectable by the addition of a particular non-zero amount of noise [4]. Accordingly, nGVS has been shown to particularly improve the perception of weak, subthreshold vestibular cues [15, 16] and vestibular-related balance function during absent (i.e., quiet standing [12]) or slow head movements (i.e., slow walking [29, 30]). In contrast, nGVS affected neither vestibular-related perception of suprathreshold cues nor balance function in the presence of dynamic and fast head kinematics in the previous studies. On the other hand, VRT particularly focuses on strong vestibular cues and rapid head movements in order to train deficient vestibular-related balance and ocular-motor functions [25]. Hence, both approaches operate on the opposing ends of the vestibular signal spectrum, which makes them unlikely to exhibit any positive or negative interference effects.

Another reason for the absence of nGVS effects on balance performance and/or confidence could be the point in time at which treatment effects were assessed in this study. Accordingly, assessment of treatment effects at T1 and T2 took place 1 day and 2 weeks after cessation of nGVS, respectively. There is so far no consensus about whether nGVS only acts during ongoing stimulation or whether it exhibits any plastic aftereffects after cessation of stimulation. In favor of the later assumption, Fujimoto et al. reported in an uncontrolled study long-term effects of nGVS on postural stability that lasted up to 6 h after cessation of stimulation [7]. Later, placebo-controlled studies did not find any evidence for long-term effects of nGVS on vestibular function [14, 20]. Irrespective of this question, our first assessment of treatment effects took place more than 12 h after the last application of nGVS and might have thus missed any potential nGVS-induced effects during ongoing stimulation or shortly after stimulation.

The observed absence of any synergistic treatment effects in the combination of VRT with nGVS does, however, not preclude a joint application of both therapeutic strategies for future treatment of BVP. As outlined above, both treatment approaches follow distinct therapeutic principles, target rather different sources of deficit in BVP, and act on differing timescales. VRT is usually applied intermittently with the aim to recruit visual and proprioceptive cues to establish a long-term sensory substitution of adaptation for vestibular impairment in BVP, while nGVS only acts during ongoing stimulation with the aim to directly improve the impaired processing of weak vestibular cues in BVP. Hence, both therapeutic options might be suitable partners for a complementary treatment strategy in BVP.
